# Experimental Approach to Moyamoya Angiopathy: Insights into Vascular Cell Crosstalk

**DOI:** 10.3390/cells15100862

**Published:** 2026-05-09

**Authors:** Gemma Gorla, Antonella Potenza, Tatiana Carrozzini, Giuliana Pollaci, Elisabetta Pasella, Erika Salvi, Isabella Canavero, Nicola Rifino, Paolo Ferroli, Marco Paolo Schiariti, Francesco Restelli, Francesco Acerbi, Anna Bersano, Laura Gatti

**Affiliations:** 1 Laboratory of Neurobiology and Cerebrovascular Unit, Fondazione IRCCS Istituto Neurologico Carlo Besta, 20133 Milan, Italy; gemma.gorla@istituto-besta.it (G.G.); antonella.potenza@istituto-besta.it (A.P.); tatiana.carrozzini@istituto-besta.it (T.C.); giuliana.pollaci@istituto-besta.it (G.P.); elisabetta.pasella@istituto-besta.it (E.P.); isabella.canavero@istituto-besta.it (I.C.); nicola.rifino@istituto-besta.it (N.R.); anna.bersano@istituto-besta.it (A.B.); 2 Department of Pharmacological and Biomolecular Sciences, University of Milan, 20122 Milan, Italy; 3 Data Science Center and Computational Multi-Omics of Neurological Disorders (MIND) Lab, Fondazione IRCCS Istituto Neurologico Carlo Besta, 20133 Milan, Italy; erika.salvi@istituto-besta.it; 4 Dipartimento di Elettronica, Informazione e Bioingegneria, Politecnico di Milano, 20133 Milan, Italy; 5 Department of Neurosurgery, Fondazione IRCCS Istituto Neurologico Carlo Besta, 20133 Milan, Italy; paolo.ferroli@istituto-besta.it (P.F.); marco.schiariti@istituto-besta.it (M.P.S.); francesco.restelli@istituto-besta.it (F.R.); 6 Department of Translational Research and New Technologies in Medicine and Surgery, University of Pisa, 56124 Pisa, Italy; francesco.acerbi@unipi.it; 7 Unit of Neurosurgery, University Hospital of Pisa, 56124 Pisa, Italy

**Keywords:** Moyamoya angiopathy, RNF213, PTP1B, cellular models, cerebrovascular diseases, EC-VSMC crosstalk

## Abstract

**Highlights:**

**What are the main findings?**

- The RNA interference approach towards RNF213 impaired angiogenesis in endothelial (EC) and vascular smooth muscle cells (VSMC), whereas the simultaneous RNF213–PTP1B silencing restored tube formation capability in EC but not in VSMC.
- A number of key angiogenesis transcripts were commonly expressed in experimental cellular models of vascular dysfunction as well as in pathological cerebral vessels from Moyamoya angiopathy patients.

**What are the implications of the main findings?**

- The relevance of RNF213 gene/protein in EC dysfunction is confirmed, with secondary effects on VSMC and vascular remodeling. Moreover, a potential and specific contribution of PTP1B phosphatase to defective angiogenesis of EC was observed, thus supporting the importance of EC–VSMC crosstalk for vascular integrity.
- A preliminary experimental setting including molecular/cellular vascular dysfunction was established, as a first step toward the development of a more advanced and representative model of Moyamoya angiopathy pathogenesis.

**Abstract:**

Background: The pathophysiological mechanisms of Moyamoya angiopathy (MA) are still largely unknown, although a dysfunctional vasculogenesis has been hypothesized to contribute to it. The association between this rare cerebrovascular condition and variants of Ring Finger Protein 213 (RNF213) strengthens the role of genetic factors in MA pathogenesis. Methods: To investigate the molecular mechanisms of MA, we carried out RNA interference (RNAi) targeting RNF213 in human endothelial cells (ECs) and vascular smooth muscle cells (VSMCs). The combined effect of RNAi and/or hypoxia on expression of key angiogenic factors was analyzed through qRT-PCR and Western blot. Functional assays were performed to characterize the impact of RNAi on vasculogenesis. Gene-expression arrays were performed on vessel walls of MA patients and controls. Results: RNF213-RNAi impaired angiogenic capability in ECs, whereas the simultaneous silencing of RNF213 and its phosphatase PTP1B restored angiogenesis function in ECs but worsened it in VSMCs. Angiogenic factor expression appeared to be modulated in ECs by the combined effects of RNAi and/or hypoxia, and in pathological vessels of MA patients as compared with controls. Conclusions: Our findings contribute to associating the relevance of RNF213 in MA cellular models and highlight the importance of EC-VSMC crosstalk for vascular integrity. Additionally, the study could lay the foundations for improving experimental models of MA pathophysiology.

## 1. Introduction

Moyamoya angiopathy (MA) is a rare cerebrovascular disorder of unknown etiology, characterized by progressive occlusion of the terminal portions of the internal cerebral arteries (ICAs). The disease onset shows a bimodal age distribution (0–10 and 40–49 years), with the adult peak characterized by a stronger female prevalence (ratio 3:1) [1,2]. The Japanese term “moyamoya” (meaning “puff of smoke”) describes the characteristic angiographic appearance of a network of fragile collateral vessels that develop to supply blood to the brain [3]. The main clinical manifestations of MA include transient ischemic attacks and ischemic strokes caused by large artery occlusion, as well as hemorrhagic strokes and aneurysms resulting from the rupture of defective compensatory vessels [4].

Histopathological examinations demonstrated that middle cerebral artery (MCA) stenosis in MA patients, differently from the classical arteriosclerotic plaque, is associated with concentric fibro-cellular intimal hyperplasia, alteration of the extracellular matrix (ECM) and proliferation of vascular smooth muscle cells (VSMCs), ultimately leading to progressive intima thickening. In contrast, the tunica media becomes thinner, and the external vessel diameter decreases progressively [5]. This process causes a lack of oxygen supply and consequent formation of a typical collateral vessel.

To date, no animal or cellular model fully recapitulated the pathological changes observed in the vascular intimal hyperplasia of MA patients. Although these models reproduce the various aspects of MA separately, none can adequately combine genetic predisposition, immunologically mediated vasculopathy, and chronic ischemia [6]. The poor understanding of MA molecular mechanisms has hindered the development of effective preventive measures or pharmacological therapies; thus, the neurosurgical procedure between MCA and superficial temporal artery (STA) remains the unique therapeutic option available for MA patients. In particular, indirect synangiosis (mostly in children) and direct extracranial-intracranial bypass represent the main surgical options [7].

The Arg4810Lys (p.R4810K) variant of the Ring Finger Protein 213 (RNF213) gene, encoding a large ATPase/E3 ubiquitin ligase, has been identified as a pathogenic trait for MA, particularly in East Asian populations [8,9]. Despite the association with MA, the precise molecular function of RNF213 in disease pathogenesis remains elusive [9]. Of note, additional environmental and genetic factors are possibly required for the disease onset because the penetrance of the pathogenic gene variant is only 1.4% in Asian populations. The RNF213 p.R4810K variant showed a key role in other vascular occlusive diseases by increasing the risk of ischemic stroke attributable to large-artery atherosclerosis [10,11,12]. Interestingly, RNF213 has been recently identified as a substrate of protein tyrosine phosphatase 1B (PTP1B), a key regulator of cellular hypoxia responses [13,14,15]. To investigate RNF213’s putative role in vascular remodeling in MA, we propose the development of a preliminary in vitro human cellular model. This experimental model aims to identify additional molecular factors involved in the crosstalk between endothelial cells (ECs) and VSMCs, which represents a key feature of MA pathophysiology.

## 2. Materials and Methods

### 2.1. HUVEC and T/G HA-VSMC Culture

HUVEC cells (ATCC^®^ CRL-1730TM) were cultured in EGM-2 medium (EGM™-2 Bullet Kit, CC-3162, Lonza Walkersville, Walkersville, MD, USA) and T/G HA-VSMCs (ATCC^®^ CRL-1999TM) were cultured in Ham’s F-12K (Kaighn’s, Thermo Fisher Scientific, Waltham, MA, USA) medium at 37 °C, 5% CO_2_. Co-cultures between HUVEC and VSMCs were maintained in a 1:1 mixture of EGM-2 and Ham’s F-12K at 37 °C, 5% CO2. All media were supplemented with 10% FBS (One Shot™, Gibco^®^ Life Technologies, ThermoFisher, Waltham, MA, USA). Cells were seeded in plates pretreated with 1 μg/cm^2^ of collagen Bornstein and Traub type IV from human placenta (Sigma-Aldrich C7521, St. Louis, MO, USA).

### 2.2. Human Sample Collection

The collection of MCA specimens after dural opening was performed by experienced neurosurgeons during the MCA-STA bypass procedure, and it consisted of a precise anastomosis aimed at improving the patient’s outcome, which is feasible only if it poses no harm to the patient [7]. A suitable amount of vessel wall fragment was obtained from a subgroup of 10 adult Caucasian MA patients. MCA specimens were also sampled in a subgroup of 5 adult Caucasian subjects (CTRL) undergoing bypass because suffering from hemodynamic cerebrovascular insufficiency due to atherosclerotic diseases or a craniotomy to clip isolated intracranial unruptured aneurysms in the anterior circulation (mostly MCA or anterior communicating artery). Only those patients who had given specific informed consent to offer specimens were recruited. Of note, this surgical revascularization procedure represents a necessary and undelayable step of the clinical practice in response to the neurological insult that determined the recourse to this therapeutic option to be of utmost importance. A multidisciplinary team consisting of neurologists, neurosurgeons and neuroradiologists discussed surgical indications for each patient and control subject, according to European MA guidelines [2]. The institutional Ethics Committee approved all the protocols of sample collection, for both MA and control patients.

### 2.3. Ethical Approval

The study design was approved by the Ethics Committee of the Fondazione IRCCS Istituto Neurologico “C. Besta” of Milan (report n° 12, 10 January 2014) and was carried out in accordance with the Declaration of Helsinki for experiments involving humans. Since it was designed as a pure observational study, patients underwent diagnostic procedures and received therapy according to local practice. Informed written consent for study participation and sample collection from all patients and controls was mandatory for study inclusion. Privacy procedures were applied to protect patients’ and controls’ personal identities.

### 2.4. RNA Interference Approaches

For RNA interference (RNAi) studies, 120,000 cells/mL HUVEC were seeded in EGM™-2, and 60,000 cells/mL T/G HA-VSMC were seeded in Ham’s F-12K with 10% FBS in 6-well plates and cultured for 24 h at 37 °C, 5% CO_2_. Silencer Select siRNA targeting two distinct regions of (s33658 and s33568, Ambion™, ThermoFisher) RNF213 mRNA (s11506 and s11507 Ambion™, ThermoFisher) and PTP1B mRNA were used. Specific scramble siRNA (Ambion™ 4390843, ThermoFisher) was used as negative control. Solution A [Opti-MEM™ (Gibco, ThermoFisher) + 20 μM scramble siRNA/negative control] and Solution B [Opti-MEM™ + Lipofectamine RNAiMAX reagent (ThermoFisher)] were mixed to obtain solution C and incubated at RT for 15 min. Opti-MEM™ medium replaced growth medium, and aliquots of solution C were added to cells and then incubated at 37 °C, 5% CO_2_. After 6 h, Opti-MEM™ was removed, and cells were incubated in their medium at 37 °C, 5% CO_2_ for 48 h. A qRT-PCR was carried out to verify the success of RNF213 or/and PTP1B RNAi.

### 2.5. Oxygen Depletion Exposure

HUVEC cells were seeded at a density of 20,000 cells/cm^2^ and cultured at 37 °C in a humidified atmosphere containing 21% O_2_ and 5% CO_2_ until reaching approximately 80% confluence. Cells were exposed for 6 h to hypoxic conditions using a Mini Galaxy E CO_2_ Incubator (RS Biotech, Irvine, UK), which allows controlled regulation of oxygen and carbon dioxide levels. Hypoxic conditions were set at 1% O_2_ and 5% CO_2_ at 37 °C in a humidified atmosphere. Normoxic controls were maintained at 21% O_2_ and 5% CO_2_. Oxygen concentration was continuously monitored and fixed by the incubator’s internal sensor to ensure stable hypoxic conditions across independent experiments. After 6 h incubation at 1% O_2_, cells were properly harvested through a scraper for subsequent analyses.

### 2.6. RNA Extraction and Quantitative Real-Time PCR Analyses in Cell Cultures

Total RNA was extracted from HUVEC and T/G HA-VSMCs using RNeasy Plus Mini Kit (Qiagen, Marshall Street, Redwood City, CA, USA) and quantified by NanoDrop (NanoPhotometer^®^ N60/N50, Implen, Westlake Village, CA, USA). RNA was reverse-transcribed with iScript Advanced cDNA Synthesis Kit (BIORAD, Hercules, CA, USA), according to the manufacturer’s protocol by Mastercycler Ep Gradient Thermal Cycler (Eppendorf, Hamburg, Germany). Quantitative Real-Time PCR (qRT-PCR) analyses were carried out by CFX-96 Real Time PCR Detection System (BIORAD). cDNA transcripts were amplified using TaqMan assays (ThermoFisher, Waltham, MA, USA) for RNF213 (Hs00326306_m1), PTP1B (PTPN1) (Hs00942477_m1), Beta-2-Microglobulin (β2M, Hs00187842_m1), and Glyceraldehyde-3-Phosphate Dehydrogenase (GAPDH, Hs99999905_m1). The relative mRNA expression was calculated through the 2^−ΔΔCt^ comparative method using β2M and GAPDH as housekeeping genes. Negative control scramble siRNA was used as the calibrator.

### 2.7. RNA Extraction from Middle Cerebral Artery (MCA) Tissue Specimens

The tissue specimens were shock-frozen in liquid nitrogen immediately after excision to maintain their structural and molecular integrity and stored at −80 °C for subsequent analysis. MCA specimens were then processed with RLT lysis buffer with 1% of β-mercaptoethanol and subjected to lysis by a tissue homogenizer (Mikro-Dismembrators, B-BRAUN, Melsungen, Germany). Proteinase-K (Euroclone, Milan, Italy) was added, and samples were incubated at 55 °C for 10 min, then centrifuged at 12,000 rpm for 3 min. Total RNA was extracted through RNeasy Fibrous Tissue Mini Kit (Qiagen, Marshall Street, Redwood City, CA, USA) according to the manufacturer’s protocol.

### 2.8. RT^2^ Profiler Array

The Human Angiogenic Growth Factors & Angiogenesis Inhibitors RT^2^ Profiler PCR Array (330231/PAXX-024Y, Qiagen) was used. Total RNA from cultured cells and from human MCA samples was reverse-transcribed with RT2 First Strand Kit (Qiagen) as reported above. Thousand ng of cDNA was mixed with RT2 SYBR Green Mastermix and placed into a 96-well array. The amplification program was 95 °C, 10 min for 1 cycle, 95 °C, 15 s and 60 °C, 1 min for 40 cycles. Analyses were performed by the CFX-96 Real Time PCR Detection System (BIORAD).

### 2.9. Western Blot Analysis

Cells were harvested and then lysed in Sample Buffer containing Tris HCl pH 6.8 (0.125 M), sodium dodecyl sulfate solution 5% (SDS; 1610416, Bio-Rad, Hercules, CA, USA), protease and phosphatase inhibitors cocktail 100-fold dilution (1861281, Thermo Fisher, Waltham, MA, USA). Micro BCA protein assay kit (23235, Thermo Fisher, Waltham, MA, USA) was used to determine protein concentration, and the same protein amount for each sample was used. Analysis was performed by SDS-PAGE electrophoresis on 4-15% Mini-PROTEAN TGX gel or 3–8% Tris-Acetate Criterion^TM^ XT PrecastGel (Bio-Rad Laboratories, Hercules, CA, USA), proteins were then transferred to a nitrocellulose membrane (BR20220117, Bio-Rad Laboratories, Hercules, CA, USA) and checked by Ponceau staining (P7170-1L, Sigma-Aldrich, St. Louis, MO, USA). Nitrocellulose membranes were blocked in PBS-Tween with 5% of powdered milk (T145.2, CARL ROTH, Karlsruhe, Germany) or Immobilon^®^ Signal Enhancer (Millipore Corporation, Billerica, MA, USA) and incubated with primary antibodies at 4 °C overnight. The following antibodies and dilution were used: anti-RNF213 [(1:250) NBP1-88466, Novus Biologicals, Centennial, CO, USA; (1:1000) NOE-TGN-201, Millipore], anti-PTP1B [(1:1000) MA5-25616, Invitrogen, Waltham, MA, USA]; anti-HIF-1α [(1:500) MA1-16504, Invitrogen]; anti-β-actin [(1:6000) VMA00048, Bio-Rad]; and anti-Vinculin [(1:1000) MA5-32759, Invitrogen]. Immunoreactive proteins were revealed by ECL (Amersham ECL Prime Western Blotting Detection reagents, RPN2232, Marlborough, MA, USA) and chemiluminescence signals were detected using CHEMIDOC MP Imaging System (Bio-Rad Laboratories, Hercules, CA, USA). The analysis was done through Image Lab 6.1 Software Bio-Rad. As a calibrator, Vinculin or β-actin was used. PTP1B and HIF-1α proteins were detected on the same membrane, which was subsequently reprobed for β-actin as a loading control. RNF213 was detected on a separate membrane, which was subsequently reprobed for vinculin as a loading control.

### 2.10. Tube Formation Assay

A Matrigel tube formation assay was done to assess HUVEC or T/G HA-VSMC angiogenic capacity in different experimental conditions (negative control, RNF213, and/or PTP1B siRNA-transfected cells). HUVECs were seeded in EGM™-2 (250 cells/μL) and T/G HA-VSMC in Ham’s F-12K (125 cells/μL) into 96-well plates at 37 °C, 5% CO_2_. Co-cultures of HUVEC (125 cells/μL) and T/G HA-VSMC (62.5 cells/μL) were maintained in a 1:1 mixture of EGM-2 and Ham’s F-12K, at 37 °C, 5% CO_2_. The 96-well plates were previously coated with Matrigel (Corning^®^ Matrigel^®^ Growth Factor Reduced, Corning, AZ, USA) and incubated (1 h, 37 °C, 5% CO_2_). Images were taken in bright field microscopy, 4× magnification, 200 μm scale bar after 16 h, and tube formation was quantified using Wimasis Image Analysis software (https://www.wimasis.com/en/WimTube, accessed on 1 February 2026; Onimagin Technologies SCA, Cordoba, Spain). At least three independent experiments, each one including three technical replicates for all the tested experimental conditions, were analyzed.

### 2.11. Statistical Analysis

The statistical significance of the single-assay mRNA expression data, calculated by the 2^−ΔΔCt^ comparative method, was determined considering the relative fold-change threshold. Upregulation was considered with 2^−ΔΔCt^  > 2 and downregulation with 2^−ΔΔCt^  < 0.5. Western blot data were expressed as mean ± SD, and statistical significance was calculated through a parametric unpaired *t*-test. Values of at least three independent experiments in triplicate were shown. Tube-forming data were analyzed through one-way ANOVA followed by Dunnett’s post hoc test to compare each treatment group with the control group (siRNA scramble). Both analyses were performed by using GraphPad Prism 8 (GraphPad Software, Inc., San Diego, CA, USA); *p*-values are schematized as follows: * < 0.05; ** < 0.01; *** < 0.001.

For RT2 Profiler PCR Array analyses the relative mRNA expression was calculated by the 2^−ΔΔCt^ comparative method. Cycle threshold (CT) values were assessed for amplification quality, and a threshold of Ct ≤ 38 was set as the cutoff for valid amplification. Targets with CT values below 15 or above 38 were excluded.

Five endogenous control genes (ACTB, B2M, GAPDH, HPRT1, RPLP0) and internal array controls (HGDC, RTC, PPC) were considered. Expression stability of endogenous controls was verified using RefFinder [16]; B2M was used for normalization after being selected as the most stable reference gene. Call rates were calculated for both the sample and the target. Samples exhibited call rates ranging from 67.5% to 96.4% across 83 valid targets. For downstream analyses, only mRNAs with a call rate ≥ 70% across samples were retained.

## 3. Results

### 3.1. RNA Interference Approaches Towards RNF213 and PTP1B in Vascular Cellular Models

**Figure 1:**
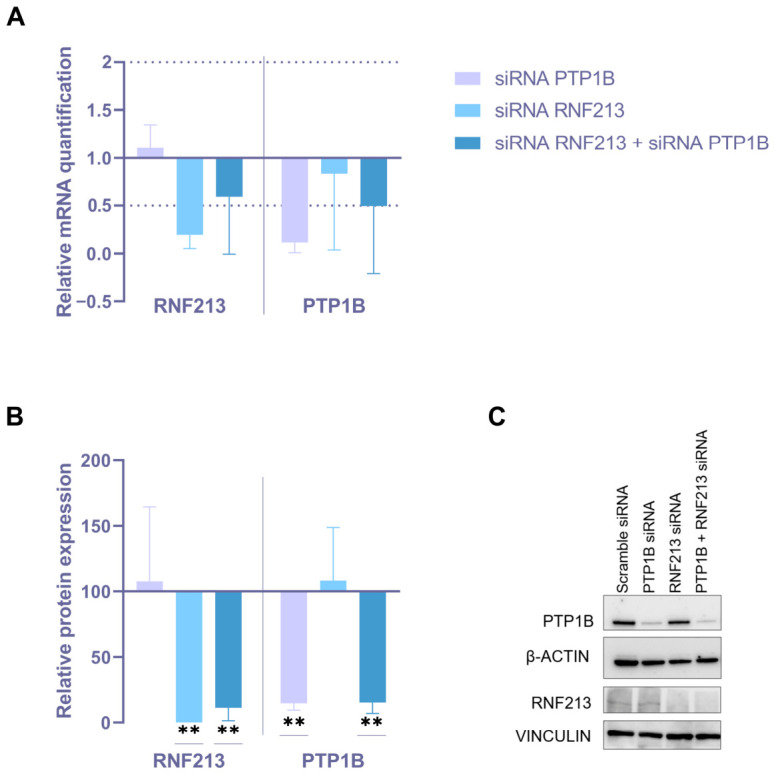
Analysis of mRNA and protein expression in endothelial cells (ECs) following RNAi targeting RNF213 and/or PTP1B. (**A**) Quantitative real-time PCR analysis of RNF213 and PTP1B mRNA expression in ECs after single or combined RNF213 and PTP1B RNAi for 48 h. Relative mRNA levels were calculated using the 2^−ΔΔCt^ method with β2M and GAPDH as housekeeping genes, and scramble siRNA was used as the calibrator. (**B**) Densitometric analysis of RNF213 and PTP1B protein expression assessed by Western blot following single or combined RNAi. β-actin and vinculin were used as loading controls for low- and high-molecular-weight proteins, respectively. Data are expressed as mean ± SD; statistical significance was assessed using an unpaired *t*-test (** *p* < 0.01). Each data point represents a biological replicate obtained from independent experiments and calculated as the mean of at least three technical triplicates. (**C**) Representative image of protein expression for each experimental condition.

**Figure 2:**
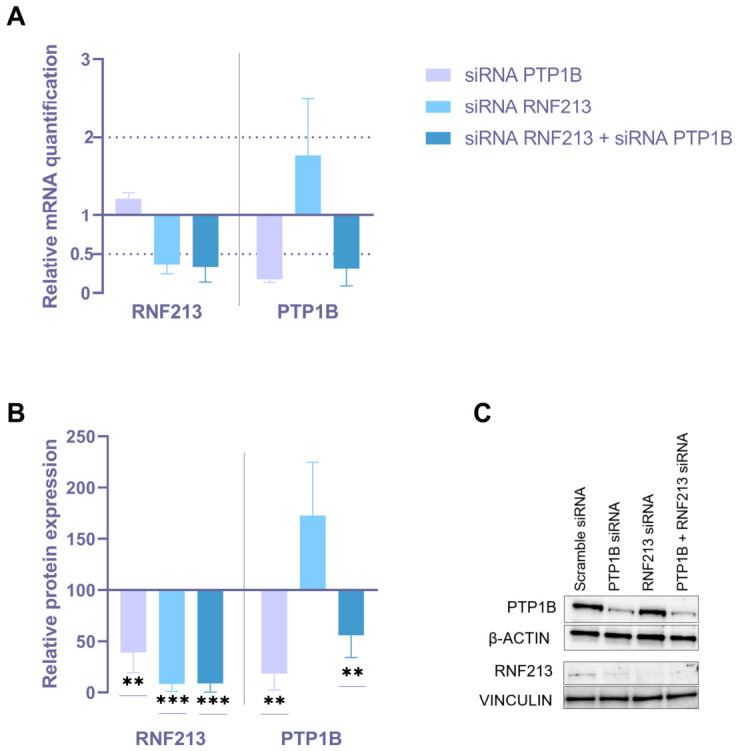
Analysis of mRNA and protein expression in vascular smooth muscle cells (VSMCs) following RNAi targeting RNF213 and/or PTP1B. (**A**) Quantitative real-time PCR analysis of RNF213 and PTP1B mRNA expression in VSMCs after single or combined RNF213 and PTP1B RNAi for 48 h. Relative mRNA levels were calculated using the 2^−ΔΔCt^ method with β2M and GAPDH as housekeeping genes, and scramble siRNA was used as the calibrator. (**B**) Densitometric analysis of RNF213 and PTP1B protein expression assessed by Western blot following single or combined RNAi. β-actin and vinculin were used as loading controls for low- and high-molecular-weight proteins, respectively. Data are expressed as mean ± SD; statistical significance was assessed using an unpaired *t*-test (** *p* < 0.01; *** *p* < 0.001). Each data point represents a biological replicate obtained from independent experiments and calculated as the mean of at least three technical triplicates. (**C**) Representative image of protein expression for each experimental condition.

Human EC and VSMC cultures underwent a transient RNA interference (RNAi) approach, by transfecting, both singularly and simultaneously, specific small interfering RNA (siRNA) towards RNF213 and PTP1B. Appropriate scramble siRNA was transfected as a negative control. After 48h from the silencing start, the analysis in ECs showed a significant downregulation of RNF213 and PTP1B mRNA and protein (RQ < 0.5 and *p* < 0.01, respectively) for all the experimental conditions, as compared to the negative control (Figure 1). Likewise, the downregulation of RNF213 and PTP1B at the mRNA and protein levels was observed in VSMCs after single/simultaneous transient transfection of the selected siRNAs (RQ < 0.5 and *p* < 0.01, respectively; Figure 2). Interestingly, VSMCs silenced for PTP1B also showed a marked downregulation of RNF213 protein expression (Figure 2B). A slight but not significant upregulation of PTP1B was found in VSMCs silenced for RNF213, at both the mRNA and the protein level (Figure 2). To clarify the underlying molecular mechanisms of RNF213-PTP1B interaction, the expression levels of other downstream key proteins were analyzed in ECs following RNAi approaches, but we did not observe significant modulations in relative protein levels (Supplementary Figure S1A–H, lanes 1–4).

### 3.2. Assessment of Tube Forming Ability in Vascular Cellular Models

**Figure 3:**
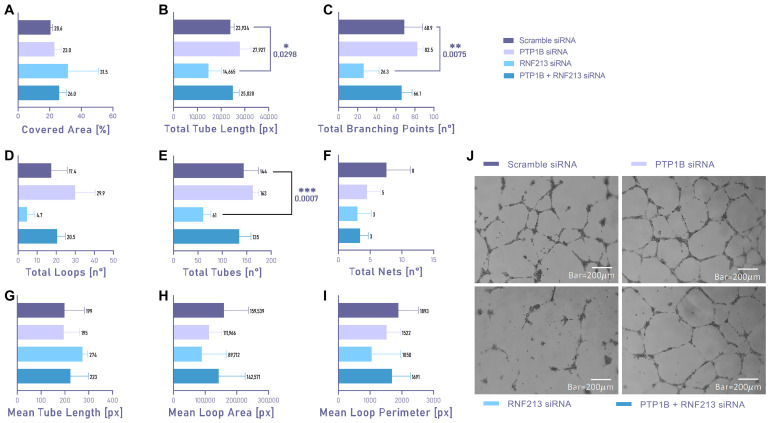
Matrigel tube formation assay in endothelial cells (ECs) following RNAi targeting RNF213 and/or PTP1B. (**A**–**I**) Angiogenic capacity of ECs was assessed for an additional 16 h following 48 h of siRNA transfection. Scramble siRNA was used as the reference condition. Data are expressed as mean ± SD; statistical significance was assessed using one-way ANOVA followed by Dunnett’s post hoc test. Statistical significance was set at *p* < 0.05 (* *p* < 0.05; ** *p* < 0.01, *** *p* < 0.001). Results from at least three independent experiments performed in triplicate are shown. (**J**) Representative bright-field microscopy images (4× magnification; scale bar, 200 μm) of tube formation in ECs transfected with scramble siRNA, RNF213 siRNA and/or PTP1B siRNA.

We performed Matrigel tube formation assays in order to assess whether RNF213 and/or PTP1B RNAi may affect cellular angiogenesis ability. RNF213-silenced ECs (Figure 3, light blue) showed impaired ability to form organized tubes as compared to the negative control condition (scramble siRNA, Figure 3, purple), as shown by the statistically significant decrease of: total Tube Length (*p* = 0.0298) (Figure 3B); total Branching Points (*p* = 0.0075) (Figure 3C); and total tubes (*p* = 0.0007) (Figure 3E). Interestingly, ECs simultaneously silenced for PTP1B and RNF213 recovered their basal vasculogenesis capacity for all the above-mentioned parameters (Figure 3, dark blue), which resulted in a condition comparable with the negative control condition. Consistently, total Tube Length, total Branching Points, total loops and total tubes were slightly increased in ECs silenced for PTP1B (Figure 3B–E, lilac) in comparison to scrambled siRNA, although without statistical significance.

**Figure 4:**
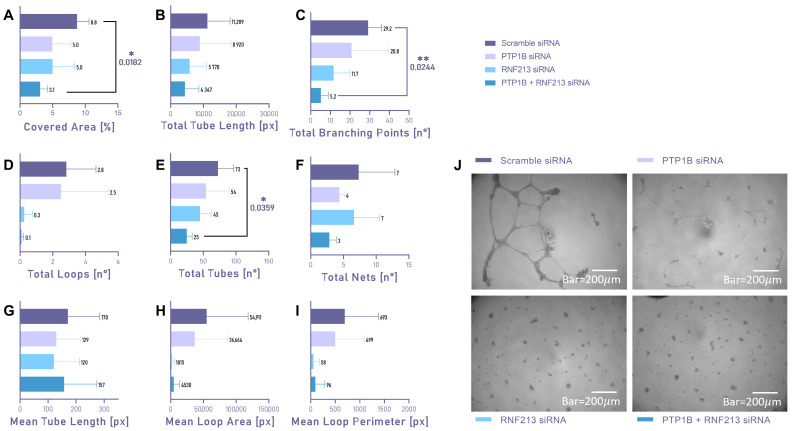
Matrigel tube formation assay in vascular smooth muscle cells (VSMCs) following RNAi targeting RNF213 and/or PTP1B. (**A**–**I**) Angiogenic capacity of VSMCs was assessed for an additional 16 h following 48 h of siRNA transfection. Scramble siRNA was used as the reference condition. Data are expressed as mean ± SD; statistical significance was assessed using one-way ANOVA followed by Dunnett’s post hoc test. Statistical significance was set at *p* < 0.05 (* *p* < 0.05; ** *p* < 0.01). Results from at least three independent experiments performed in triplicate are shown. (**J**) Representative bright-field microscopy images (4× magnification; scale bar, 200 μm) of tube formation in VSMCs transfected with scramble siRNA, RNF213 siRNA and/or PTP1B siRNA.

VSMCs transfected with both RNF213 and PTP1B siRNA simultaneously further worsened the ability to organize tubes in comparison with scramble siRNA, concerning Covered Area (*p* = 0.0182), total Branching Points (*p* = 0.0244), and total tubes (*p* = 0.0359) (Figure 4A,C,E, dark blue). A slight decrease in the tube-forming parameters is appreciable in VSMC silenced for RNF213 and PTP1B alone, although without statistical significance (Figure 4, light blue and lilac). To investigate the reciprocal cross-talk between ECs and VSMCs, a Matrigel tube formation assay was also performed in ECs co-cultured with VSMCs, following RNF213 RNAi. Specifically, we found that ECs seem to have a higher baseline ability to form tubes compared to VSMCs, in terms of Covered Area (*p* = 0.0133) and Mean Loop Perimeter (*p* = 0.0479). Scramble-siRNA-transfected EC–VSMC co-cultures have a reduced ability to form tubes, particularly in terms of Mean Loop Area (*p* = 0.0432) and Mean Loop Perimeter (*p* = 0.0267). (Supplementary Figure S2).

### 3.3. Combined Effects of RNAi Approaches and Hypoxia Stress in ECs

**Figure 5:**
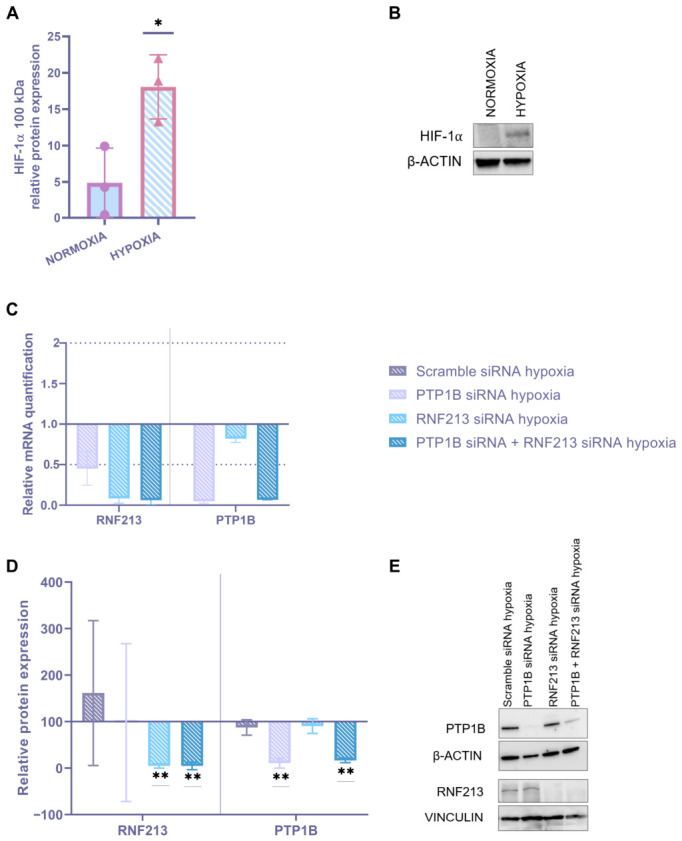
Gene and protein expression analysis in endothelial cells (ECs) following RNAi targeting RNF213 and/or PTP1B under hypoxic stress. (**A**) Densitometric analysis of HIF-1α protein expression in scramble siRNA–transfected ECs after 6 h of 1% O_2_. β-actin was used as the loading control. Data are expressed as mean ± SD; statistical significance was assessed using an unpaired *t*-test (* *p* < 0.05). (**B**) Representative image of protein expression. (**C**) Quantitative real-time PCR analysis of RNF213 and PTP1B mRNA expression in ECs after transient RNF213 and/or PTP1B siRNA transfection for 48 h followed by 6 h of 1% O_2_. Relative mRNA levels were calculated using the 2^−ΔΔCt^ method with β2M and GAPDH as housekeeping genes, and scramble siRNA was used as the calibrator. (**D**) Densitometric analysis of RNF213 and PTP1B protein expression assessed by Western blot. β-actin and vinculin were used as loading controls for low- and high-molecular-weight proteins, respectively, and the scramble siRNA condition under normoxia (i.e., 21% O_2_) was used as the calibrator. Data are expressed as mean ± SD; statistical significance was assessed using an unpaired *t*-test (** *p* < 0.01). Each data point represents a biological replicate obtained from independent experiments and calculated as the mean of at least three technical triplicates. (**E**) Representative image of protein expression.

Since hypoxia-induced endothelial dysfunction seems to be a critical aspect of MA pathogenesis, we evaluated the combined effects of RNAi approaches and hypoxia in ECs, which are considered the primary sensor responding to oxygen depletion. Preliminarily, the expression of the active form of HIF-1α protein (MW ~100 kDa) was evaluated in scramble siRNA ECs after 6h oxygen deprivation (i.e., 1% O_2_) to confirm the induction of a cellular response to hypoxic stress. As expected, HIF-1α protein expression was significantly increased as compared to scramble siRNA ECs in normoxia (i.e., 21% O_2_) conditions (Figure 5A,B).

Subsequently, to evaluate the persistence of RNAi in ECs after 6h oxygen depletion, we analyzed the modulation of RNF213 and/or PTP1B at the mRNA level (Figure 5C) and at protein expression (Figure 5D). Specifically, we observed a significant downregulation of both RNF213 and PTP1B mRNA and protein (RQ < 0.5 and *p* < 0.01, respectively) for all the RNAi experimental conditions, as compared to the negative control (scramble siRNA) one.

As expected, oxygen depletion acted as a critical pathological stimulus on VSMCs, driving them to a phenotypic shift leading to massive cell death.

To clarify the combined molecular effects elicited by RNAi approaches and hypoxia stress in ECs, the expression levels of other downstream key proteins were analyzed following RNF213/PTP1B siRNA transfection and oxygen depletion (Supplementary Figure S1A–H, lanes 5–8). Specifically, we confirmed the hypoxia-induced upregulation of HIF1α in all the tested experimental conditions.

### 3.4. Gene Expression Analysis of Key Angiogenic Factors in ECs Following RNAi Approaches and Hypoxic Stress

**Figure 6:**
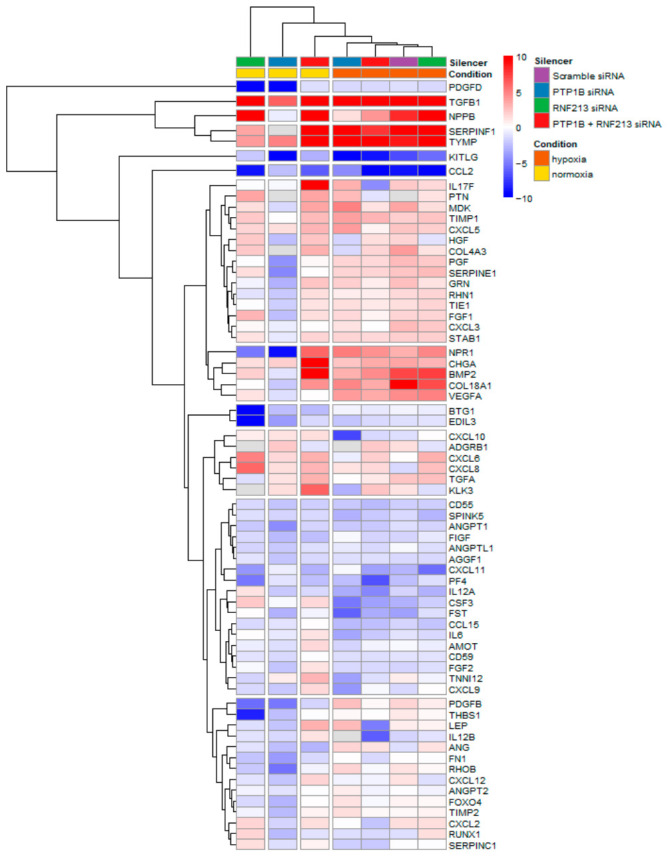
RT^2^ Profiler PCR Array analysis on experimental MA cellular models. To assess differential gene expression of Human Angiogenic Growth Factors and Angiogenesis Inhibitors, ECs were transfected with selected siRNAs for 48 h and then exposed to 1% O_2_ for 6 h. The heatmap shows mean fold change (FC) values for genes with a call rate ≥70% across all samples. Columns represent experimental conditions, and rows represent analyzed gene targets. Categorical annotations are shown as color-coded bars above the heatmap, indicating the oxygen percentage (orange for hypoxia, 1% O_2_; yellow for normoxia, 21% O_2_) and siRNA transfection (purple for scramble siRNA, blue for PTP1B siRNA, green for RNF213 siRNA, and red for PTP1B-RNF213 siRNA).

RT^2^ Profiler Arrays were used to evaluate the modulation of gene expression (GE) induced by the combined effect of RNF213 and/or PTP1B RNAi and hypoxic stress (1% O_2_) in ECs (Figure 6 and Supplementary Table S1). The differential expression profile of 84 “Human Angiogenic Growth Factors and Angio genesis Inhibitors” transcripts was determined based on fold change values (2^−ΔΔCT^ method) and reported by a heatmap, by using the scramble siRNA/normoxia (i.e., 21% O_2_) condition as the calibrator within each experimental batch. For each experimental condition, the mean fold change was computed across three independent experiments. Notably, Platelet-Derived Growth Factor D (PDGF-D) and Transforming Growth Factor beta-1 (TGF-β1) were found as the two main deregulated genes. Specifically, PDGF-D showed a very significant downregulation in RNF213 siRNA and PTP1B siRNA under normoxia conditions and in all hypoxic conditions (RQ < 0.5), while TGF-β showed a very significant upregulation for all the silenced conditions (RQ > 1), as compared to the negative control one (Figure 6, Supplementary Table S1).

### 3.5. Gene Expression Analysis of Key Angiogenic Factors in MCA Specimens from MA Patients

**Figure 7:**
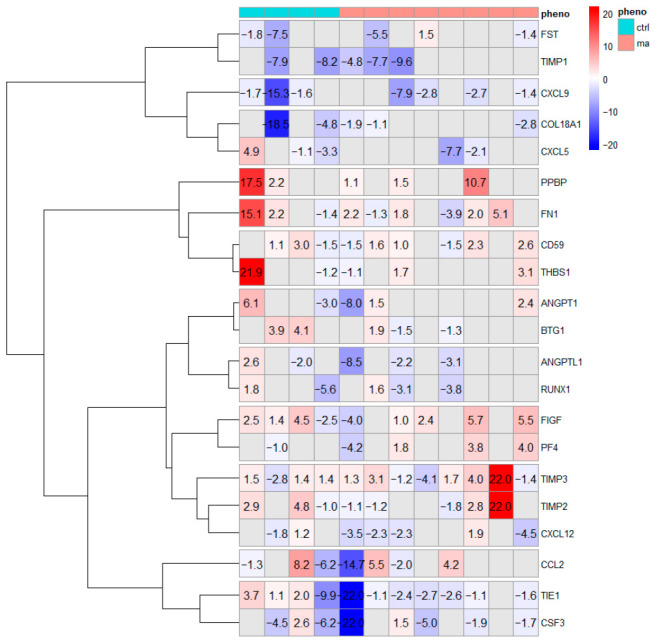
RT^2^ Profiler PCR Array analysis on MA tissues. To assess differential gene expression of Human Angiogenic Growth Factors and Angiogenesis Inhibitors, MCA specimens from MA patients were compared to MCA fragments from unrelated subjects (CTRL) who underwent an STA-MCA neurosurgical bypass procedure. The heatmap shows mean fold change values for genes with a call rate ≥70% across all samples. Columns represent samples from 8 different MA patients (red) and 4 CTRL (blue), and rows represent the resulting modulated transcripts.

The mRNA modulation of “Human Angiogenic Growth factors and Inhibitors” was also evaluated in MCA pathological samples collected from MA patients (n = 10) and age/sex-matched control subjects (CTRL, n = 5), during the STA-MCA neurosurgical bypass procedure [7]. Thirty-five out of the 84 analyzed genes were detectable (49 transcripts gave nonspecific expression in MCA specimens), whereas 14 others were excluded after statistical analysis because they were considered unreliable, leading to 21 transcripts considered for the analysis. GE analysis showed a distinct angiogenic profile in MA patients compared to controls (CTRL), as shown by hierarchical clustering (Figure 7).

**Figure 8:**
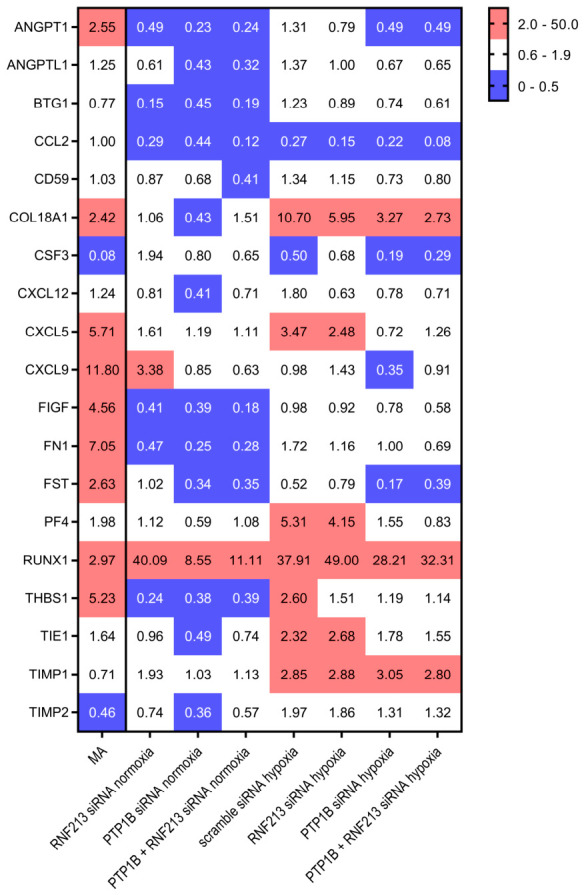
RT^2^ Profiler PCR Array analysis on MCA samples from MA patients and MA experimental cellular models. Fold change (FC) values by RT^2^ Profiler PCR Array are reported for 19 transcripts expressed at detectable levels both in MCA specimens from MA patients (first column) and endothelial cells (ECs) in response to RNAi with/without hypoxic stress. Relative quantification was performed using the 2^−ΔΔCt^ method, where ΔΔCq represents the difference between normalized expression levels (ΔCq). MCA specimens collected from control subjects were chosen as calibrators for GE analysis in tissues. The negative control scramble siRNA condition was considered as a calibrator for experimental cellular models. FC values were calculated as 2^−ΔΔCt^, and negative inverse values were used to indicate FC reduction. Downregulated transcripts (i.e., 2^−ΔΔCt^  < 0.5) are included in blue boxes, and upregulated ones (i.e., 2^−ΔΔCt^ > 2) are in red boxes, respectively.

Of the 84 transcripts tested by RT^2^ Arrays, 19 were detectable in both the GE analyses, performed on MCA samples from MA patients, as well as on ECs under RNAi/hypoxia conditions, respectively (Figure 8). Some key genes, such as Runt-Related Transcription Factor 1 (RUNX1) and Collagen Type XVIII Alpha 1 Chain (COL18A1), showed expression changes similar to those observed in ECs exposed to RNAi and hypoxic stress. Other genes exhibited patterns resembling ECs exposed to hypoxia and RNF213 silencing, particularly C-X-C motif chemokine ligand 5 (CXCL5), whereas colony-stimulating factor 3 (CSF3) showed a comparable expression profile following PTP1B silencing, both alone and in combination with RNF213. C-X-C motif chemokine ligand 9 (CXCL9) displayed expression patterns similar to those observed in ECs subjected only to RNF213 silencing.

## 4. Discussion

The lack of reliable cellular and animal models has substantially hindered the elucidation of molecular mechanisms driving MA pathogenesis [6]. This gap of knowledge represents a major obstacle in the way of developing targeted therapeutics as an effective cure for MA patients.

Targeted RNAi approaches towards critical molecular players and oxygen depletion assays were carried out to reproduce experimental cellular conditions potentially resembling the MA pathophysiological context. Specifically, we focused on gene/protein expression and vasculogenesis response of ECs and VSMCs, which are the vascular components previously implicated in MA defective angiogenesis [14,17,18].

In this experimental condition, ECs undergoing RNF213 RNAi showed a marked impairment in their angiogenic ability. Similarly, it has been reported that ECs carrying the RNF213 p.R4810K variant and exposed to oxygen-glucose deprivation exhibited endothelial dysfunction [17]. In addition, in stable RNF213-deficient human cerebral microvascular ECs, it has been demonstrated that RNF213 could be a key regulator of cerebral endothelium integrity, whose disruption could be an early pathological mechanism leading to MA [18]. The loss of RNF213 was also associated with enhanced, yet still dysfunctional, angiogenesis in experimental cell models, both in vivo and in vitro [19].

Interestingly, we observed that EC vasculogenic ability was restored when both RNF213 and its newly identified phosphatase PTP1B were simultaneously downregulated. To date, a precise characterization of RNF213-PTP1B interaction remained unaccomplished in MA, due to the cell-type specificity of protein function. Accordingly, the RNAi-induced effects in ECs appeared strongly cell-context dependent, whereas the simultaneous mRNA silencing of RNF213 and PTP1B in VSMCs caused an impairment of angiogenesis capability. Notably, PTP1B siRNA in VSMCs also downregulated RNF213 protein expression, consistent with previous in silico studies reporting that PTP1B inactivation reduced RNF213 levels [20]. Nevertheless, PTP1B seems to be relevant to MA and more generally to cerebrovascular diseases, as several studies showed that its inhibition reduces cerebrovascular injury in post-stroke recovery [21,22,23].

Relevant studies carried out in tumor cells by Neel and colleagues, which focused on the established physical interaction between RNF213 and its phosphatase PTB1B, are in agreement with our findings in normal cells [13,14,15]. The first investigation on the PTP1B–RNF213 axis was carried out in hypoxic tumor cells and revealed that PTP1B deficiency promoted RNF213-dependent ubiquitination and degradation [13]. Further mechanistic insights showed that PTP1B dephosphorylated RNF213, influencing its oligomerization and activation. Loss of PTP1B seems to activate RNF213, thus inhibiting tumor cell proliferation under hypoxic stress [14,15].

Although MA is clinically characterized by chronic cerebral hypoperfusion, cerebral blood flow is highly dynamic and associated with complex and unstable flow patterns, as well as recurrent transient ischemic events [24]. These features suggest that, at the cellular level, hypoxic exposure is likely to be intermittent and fluctuating rather than continuously sustained, supporting the use of acute or short-term hypoxic stimuli in in vitro models. Oxygen depletion has been applied as an acute condition by short-term exposures in cellular models to investigate its impact on MA, specifically through the dysfunction of the RNF213 gene [17].

In the current experimental setting, the modulation of GE following RNAi and hypoxic stress in ECs was suggestive of the typical MA vascular dysfunction. Of note, candidate targets were prioritized based on fold change, and only the most strongly modulated targets were considered. ECs, as primary sensors of blood flow and oxygen levels, typically react to hypoxia by inducing pro-angiogenic genes and initiating crosstalk with neighboring VSMCs, a critical step in vascular remodeling. Accordingly, the main modulated genes are involved in the EC-VSMC crosstalk. Specifically, PDGF-D and TGF-β1, which showed the most significant downregulation and upregulation, respectively, are two potent transforming and angiogenic growth factors secreted by ECs in response to shear stress and play a central role in EC-VSMC crosstalk, as well as vascular remodeling, by driving VSMC migration and proliferation [25,26].

EC-VSMC communication occurs both through direct cell–cell contact and via the ECM, soluble factors, and extracellular vesicles [27]. Hypoxia-related signaling results in the release of cytokines, chemokines and growth factors, which can cause EC dysfunction and disturb EC-VSMC crosstalk. Such a perturbation affects the morphology of VSMCs, which, in response to vascular injury, inflammation, or metabolic stress, can undergo phenotypic switching, transforming from a contractile to a synthetic/proliferative phenotype [28]. The transition results in excessive VSMC proliferation and migration, together with reduced expression of contractile proteins, vascular wall thickening and luminal narrowing, ultimately leading to hypertension and vascular remodeling [29,30,31].

Our results, obtained in elaborated cellular models but still inadequate to represent the complexity of the clinical–pathological condition of MA, are, however, widely supported by similar analyses conducted on valuable tissue specimens of extreme rarity. Indeed, MCA pathological samples from MA patients displayed a peculiar GE profile as compared to corresponding tissues from unrelated individuals that underwent an analogous bypass procedure. More interestingly, we observed a partial overlap in GE of key vascular regulators between MA MCAs and RNAi-ECs (e.g., CXCL5; CSF3; CXCL9). Other transcripts, such as those encoding for RUNX1 and COL18A1, exhibited expression patterns that were more consistent with those observed in ECs exposed to both RNAi and oxygen depletion, suggesting the relevance of hypoxia-related signaling pathways in MA-associated vascular alterations.

These findings collectively suggested a complex and multifactorial dysregulation of angiogenic signaling in MA, potentially involving both intrinsic endothelial dysfunction and adaptive responses to microenvironmental stressors. However, further functional and mechanistic studies will be necessary to clarify the precise role of these molecular changes and to determine their contribution to MA pathophysiology. Importantly, cerebral vessels in MA patients are free of atherosclerotic plaques, which differentiates them from what is typically seen in sporadic stroke cases [10]. Instead, it has been suggested that wall thickening and stenosis in MA is caused by VSMC proliferation, migration and invasion of the intima of the terminal ICA. This process may accord with the hypothesis of wall shear stress (WSS) as a key pathogenic driver. Computational fluid dynamics models have shown that elevated WSS values occur in the terminal ICA of MA patients, likely damaging ECs and inducing a VSMC-mediated repair response, leading to intimal hyperplasia [31]. Intriguingly, later studies proposed that increased flow velocity may serve as a trigger in MA pathogenesis. Elevated velocity has been observed not only in stenotic but also in non-stenotic ICAs of MA patients, offering insight into disease susceptibility and lesion formation mechanisms [32,33].

Several study limitations should be acknowledged. Firstly, the cell types used in this work did not fully resemble the unique features of cerebral vascular cells. Indeed, to enhance physiological relevance, studies are increasingly adopting human-induced pluripotent stem cell (iPSC)-derived brain microvascular ECs, which better simulate blood–brain barrier characteristics. Furthermore, constructing multicellular, 3D microfluidic models that include brain-specific astrocytes, pericytes, and cerebral VSMCs will be crucial to modeling the true NVU interaction. A second limitation is the inability to model systemic conditions and the poor tissue heterogeneity. To address these limitations, future studies could focus on advanced CRISPR/Cas9 technology to set up a more stable and reliable experimental model of RNF213 loss of function. On the other hand, the simplification and isolation of complex processes can facilitate their systematic investigation and deeper understanding. A further limitation of this study is that, given the limited sample size and the exploratory nature of the array analysis, applying standard statistical tests with multiple testing correction was not feasible without a substantial loss of sensitivity. We think that, despite the limited sample size, the study retains strong scientific relevance, as the rarity of the analyzed samples makes their sourcing extremely difficult. Given the preliminary and exploratory nature of the research, the results should not be considered conclusive but rather aim to generate new biological hypotheses that will need to be tested on larger cohorts.

Overall, our findings suggested that RNF213 imbalance might induce EC dysfunction, with secondary effects on VSMC and vascular remodeling. In addition, we observed that disturbance of the RNF213–PTP1B axis impaired angiogenesis, contributing to vascular instability. Moreover, we suggested that the disturbance of EC-VSMC communication may be pivotal to MA onset. The study represents an effort to dissect molecular pathways involved in MA pathogenesis by taking advantage of human vascular cellular models. While limited, our preliminary approach enables a focused investigation into the expression and function of selected key factors. Ultimately, it lays the groundwork for the improvement of experimental models, including patient-derived primary cells, miniature tissues, or human 3D primary cell models, which are increasingly considered more representative than animal models, especially for neurological diseases or species-specific cellular phenotypes [34,35].

## 5. Conclusions

It is increasingly evident that the pathophysiology of MA encompasses many different mechanisms and that the use of experimental models will refine and improve our understanding of the angiopathy. This pilot study does not claim to represent a reliable MA model, but it could establish a first step toward the development of more advanced and physiologically representative systems. The first step would be the validation of our results on more accurate and complex brain vascular cellular models, as mentioned above. Then, the identification of additional components of the RNF213-PTP1B axis could help our understanding of the interaction between RNF213 and other angiogenesis regulators in MA pathophysiology. Furthermore, given the limited availability and the preciousness of biological samples, it would be worthwhile to perform more extensive proteomics/metabolomics analyses in order to simultaneously analyze a much broader spectrum of proteins. In conclusion, our findings, strengthened by broader and prospective studies, could achieve the goal to identify potential associated factors or therapeutic targets for personalized care of MA patients.

## Data Availability

The original contributions presented in this study are included in the article/Supplementary Materials. Further inquiries can be directed to the corresponding author.

## Supplementary Materials

The following supporting information can be downloaded at: https://www.mdpi.com/article/10.3390/cells15100862/s1, Table S1: Fold-change values for expression of key angiogenesis transcripts in EC response to RNAi approaches and hypoxia stress; Fgure S1: Modulation in realative protein levels of downstream key factors; Figure S2: Matrigel tube formation assay in endothelial cells (ECs)–vascular smooth muscle cells (VSMCs) co-culture following RNF213 RNAi.

## Abbreviations

The following abbreviations are used in this manuscript:

EC	Endothelial Cell
ECM	Extracellular Matrix
MA	Moyamoya Angiopathy
MCA	Middle Cerebral Artery
NVU	Neurovascular Unit
PTP1B	Protein Tyrosine Phosphatase 1B
RNAi	RNA interference
RNF213	Ring Finger Protein 213
siRNA	Silencer RNA
STA	Superficial Temporal Artery
VSMC	Vascular Smooth Muscle Cell
