# Supplementary material for: Experimental Approach to Moyamoya Angiopathy: Insights into Vascular Cell Crosstalk
Source: Cells. 2026 May 9;15(10):862. doi: 10.3390/cells15100862 (PMC13204289; doi:10.3390/cells15100862)
Supplement: Supplementary file 1 [file cells-15-00862-s001.zip › cells-4262494-supplementary.pdf]

Table S1. Fold-change values for expression of key angiogenesis transcripts in EC response to RNAi approaches and hypoxia stress [a].

Target	RNF213 siRNA normoxia	PTP1B siRNA normoxia	PTP1B + RNF213 siRNA normoxia	scramble siRNA hypoxia	RNF213 siRNA hypoxia	PTP1B siRNA hypoxia	PTP1B + RNF213 siRNA hypoxia
ANG	-1.051	-2.574	-2.936	-1.219	1.065	1.673	1.064
ANGPT1	-2.024	-4.436	-1.760	-1.225	-1.683	-2.192	-2.078
ANGPT2	-0.405	-1.072	-0.148	-0.106	-0.422	0.726	-0.428
ANGPTL1	-1.643	-2.197	-1.371	-0.237	-1.339	-0.976	-1.584
BTG1	-15.921	-2.571	-2.763	-0.426	-0.648	-0.490	-0.956
CCL15	-1.403	-1.130	0.024	-1.710	-2.329	-2.682	-2.402
CCL2	-8.806	-2.419	-6.239	-8.883	-16.503	-4.495	-15.334
CD55	-1.465	-2.392	-1.899	-1.805	-1.916	-2.074	-2.771
CD59	-1.114	-1.408	-0.049	-1.187	-1.153	-1.343	-1.289
COL18A1	0.108	-2.181	4.342	10.004	6.820	4.657	3.317
CSF3	2.023	-0.341	1.524	-3.187	-1.950	-5.224	-3.742
CXCL10	0.767	1.179	1.228	-1.246	0.164	-7.193	-1.563
CXCL11	-4.042	-0.693	-3.043	-2.896	-5.783	-0.371	-3.776
CXCL2	1.604	-1.760	1.352	1.307	1.230	-0.194	-2.379
CXCL3	0.299	-0.610	0.152	2.673	2.055	1.172	0.099
CXCL6	4.846	1.508	3.049	0.026	3.149	-0.715	1.918
EDIL3	-14.186	-3.927	-1.500	-1.224	-1.196	-2.365	-1.406
FGF2	-0.209	-2.312	1.177	-1.667	-1.354	-1.441	-1.517
FN1	-2.294	-3.979	-1.583	0.097	-0.164	0.041	-1.508
FOXO4	-1.443	-2.892	0.038	0.516	0.272	1.085	-0.225
FST	0.084	-3.002	-0.402	-3.683	-1.210	-6.127	-3.352
GRN	-0.590	-3.159	2.370	2.531	1.033	1.925	0.605
IL6	0.091	-0.849	1.126	-1.183	-1.470	-3.431	-2.167
KITLG	-2.023	-43.772	-3.145	-6.881	-5.646	-12.750	-8.840
MDK	1.388	-1.583	3.574	3.479	1.299	4.880	1.141
NPR1	-5.240	-9.098	5.964	3.128	4.740	5.195	4.210
PDGFB	-5.794	-5.336	-1.663	1.799	0.745	2.501	0.222
PDGFD	-16.763	-9.883	-1.345	-1.273	-1.588	-1.483	-1.529
PGF	-0.096	-4.282	0.303	2.755	2.012	1.632	1.498
RHN1	-1.061	-2.158	1.377	1.550	1.775	0.665	0.631
RHOB	-2.022	-5.476	-0.505	1.107	0.272	1.792	-0.240
SERPINE1	1.248	-4.666	0.137	2.202	2.689	1.628	1.606
STAB1	1.083	-0.846	1.614	1.859	1.972	1.696	1.582
TGFB1	16.939	6.137	16.467	21.637	23.410	25.545	26.985
THBS1	-8.404	-2.489	-0.152	0.896	0.225	0.282	0.176
TIE1	-0.012	-1.954	1.041	1.296	1.664	1.228	0.883
TIMP1	2.005	0.183	2.664	1.804	2.249	3.749	2.982
TIMP2	-0.489	-2.621	0.437	0.364	0.566	1.362	0.341
AGGF1	-1.165	-2.292	-1.526	-1.432	-1.410	-1.339	-1.350
AMOT	-0.754	-1.266	1.526	-0.288	-0.711	-1.787	-0.406
CXCL12	-1.195	-2.388	1.785	1.133	-1.283	-0.484	-0.558
CXCL5	1.680	1.296	2.855	2.296	1.917	3.945	0.584
CXCL8	5.938	1.329	2.969	-1.454	2.532	1.042	1.485
IL12A	1.072	-2.117	-1.459	-1.770	-3.170	-3.214	-4.689
IL17F	-0.086	-0.392	10.097	2.033	1.405	3.117	-4.461
LEP	-1.211	-2.316	3.104	0.656	0.459	2.694	-5.008

RUNX1	1.670	-2.639	-1.064	-1.214	1.223	-1.329	-1.548
SPINK5	-1.214	-1.705	-1.505	-1.454	-2.809	-3.010	-2.114
VEGFA	1.064	-1.328	0.023	4.535	4.885	3.972	3.376
CHGA	1.376	1.994	9.692	3.440	2.858	2.313	3.376
FIGF	-1.404	-2.643	-2.497	-1.662	-0.822	-0.354	-1.786
HGF	2.056	-2.445	2.219	1.613	-1.197	-1.625	1.347
NPPB	13.361	-0.641	20.226	8.125	9.758	1.395	4.036
PF4	-5.389	-1.035	-2.479	-2.585	-1.310	-2.514	-6.727
SERPINF1	3.482	N/A	13.177	12.817	12.996	9.849	7.621
TGFA	-1.338	1.110	3.127	2.242	2.406	0.038	0.847
TNNI12	-1.693	0.604	2.885	0.692	-0.509	-3.646	-1.320
COL4A3	2.173	N/A	3.117	3.918	1.197	-1.454	1.693
SERPINC1	1.257	-1.385	0.507	-0.167	-0.057	-1.828	-2.129
TYMP	3.983	4.857	18.896	8.649	18.947	10.754	14.520
ADGRB1	N/A	2.144	-1.028	1.214	-1.064	N/A	2.042
BMP2	1.866	-1.133	10.126	7.111	7.362	3.117	4.469
CXCL9	-1.424	-2.085	1.499	-1.454	0.124	-4.028	-0.204
FGF1	2.828	-2.412	1.257	1.693	1.717	1.429	0.476
IL12B	-1.014	-1.414	1.141	-1.790	-1.165	N/A	-6.320
KLK3	N/A	1.357	6.063	1.042	-1.231	-3.053	2.362
PTN	3.458	N/A	3.411	N/A	1.028	2.732	-1.035

[a] RT^2 Profiler PCR Array analysis. To assess differential gene expression of Human Angiogenic Growth Factors and An-giogenesis Inhibitors ECs were transfected with selected siRNAs for 48 h and then exposed to 1% O_2 for 6 h.

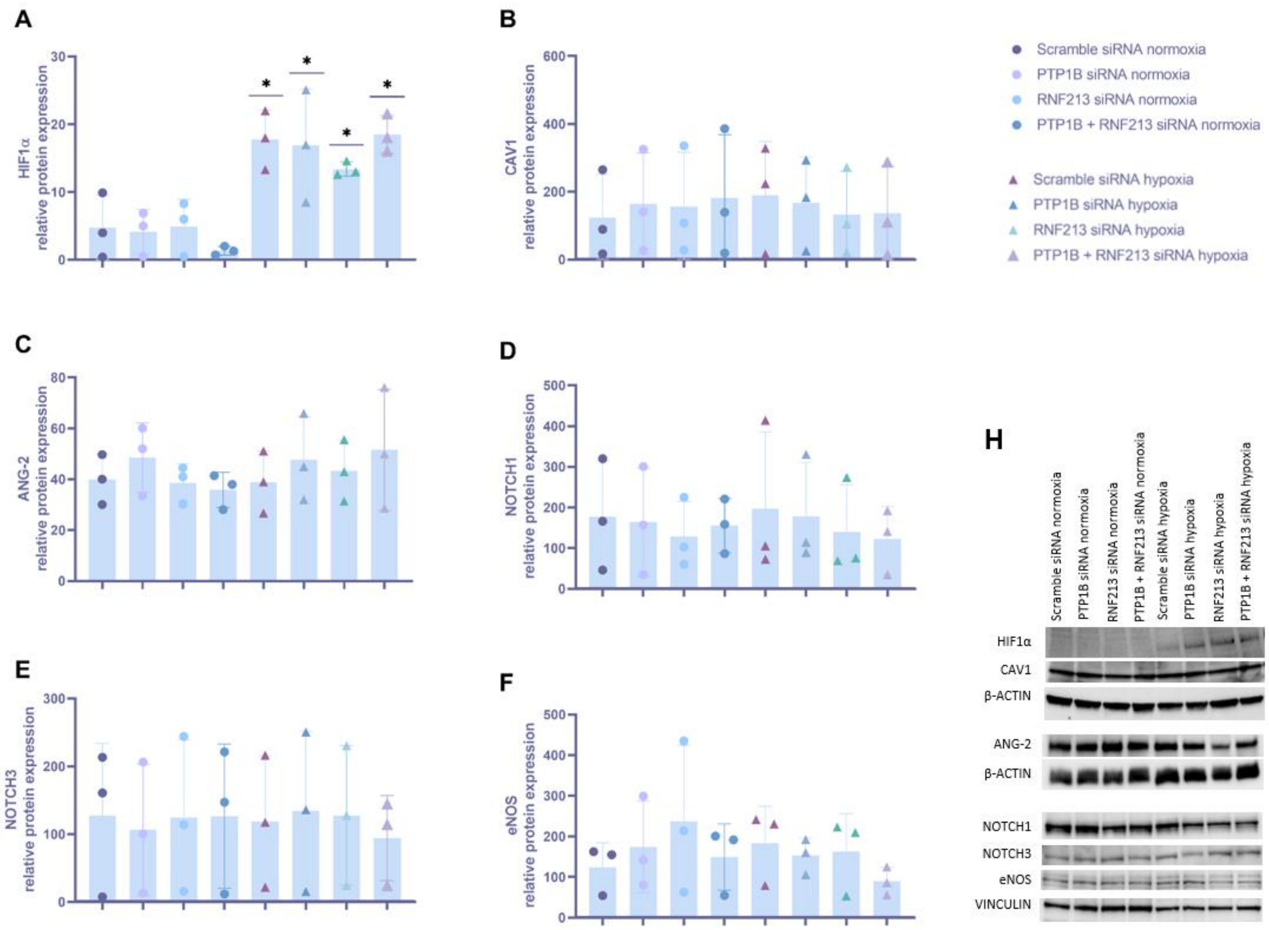

Figure S1. Modulation in realative protein levels of downstream key factors. Densitometric analysis of HIF1α, CAV1, ANG-2, NOTCH1, NOTCH3, and eNOS protein expression in endothelial cells (ECs) assessed by Western blot following single or combined RNAi following RNAi targeting RNF213 and/or PTP1B, under normoxic and hypoxic conditions. β-actin and vinculin were used as loading controls for low- and high-molecular-weight proteins, respectively. Densitometric values were normalized to the scramble siRNA under normoxic conditions. Data are expressed as mean ± SD; statistical significance was assessed using an unpaired t-test (*$p < 0.05$). Each data point represents a biological replicate obtained from independent experiments and calculated as the mean of at least three technical replicates. (H) Representative images of protein expression for each experimental condition, in normoxia (lanes 1-4) and hypoxia (lanes 5-8), respectively.

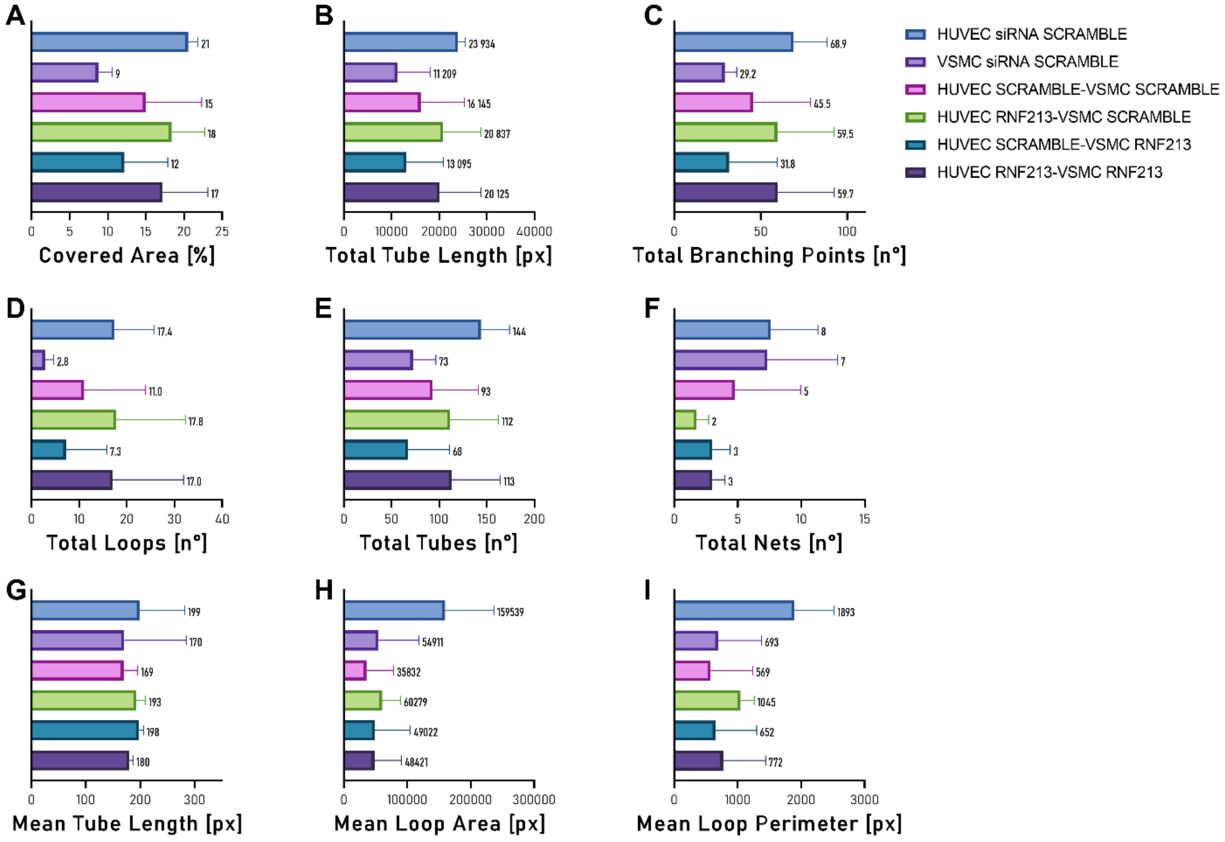

Figure S2. Matrigel tube formation assay in endothelial cells (ECs)–vascular smooth muscle cells (VSMCs) co-culture following RNF213 RNAi. (A–I) The angiogenic capacity was assessed over additional 16 h following 48 h of siRNA transfection. Scramble siRNA was used as the reference condition. Data are expressed as mean ± SD. Statistical significance was assessed using one-way ANOVA followed by Dunnett's post hoc test. A p-value < 0.05 was considered statistically significant. Results from at least three independent experiments, each performed in triplicate, are shown.
